# Validamycin Ingestion: A Rare Case of Poisoning With a Surprisingly Benign Outcome – Our Experience in a Resource‐Limited Emergency Setting

**DOI:** 10.1002/ccr3.70951

**Published:** 2025-10-03

**Authors:** Anish Luitel, Sweekar Dahal, Kabita Yadav, Bodhraj Budhathoki, Chandan Chaudary, Rasmita Poudel

**Affiliations:** ^1^ Department of Emergency Medicine Municipal Hospital Lalbandi Sarlahi Nepal; ^2^ Department of Psychiatry Municipal Hospital Lalbandi Sarlahi Nepal; ^3^ District Hospital Chautara Sindhupalchowk Nepal

**Keywords:** case report, poisoning, resource‐limited setting, symptomatic management, Validamycin

## Abstract

Validamycin ingestion is a rare encounter in emergency settings. There is no specific antidote, with management being largely symptomatic. Overzealous treatment and referral to higher centers are not necessary, as consumption of even large doses of Validamycin is non‐toxic to humans; rather, addressing the intention behind ingestion is more important.

## 1 Introduction

Poisoning is one of the major public health concerns worldwide [1]. It is a common medical emergency with significant mortality and morbidity. According to the World Health Organization, the most common cause of poisoning is intentional self‐harm [2]. In agrarian countries like Nepal, pesticides are the most frequently chosen means of suicide, with organophosphates being the most commonly encountered toxin [3, 4]. However, healthcare providers occasionally come across compounds that are unfamiliar in terms of their pharmacological properties and clinical effects. Here, we present a case of a patient who ingested a large amount of Validamycin and was successfully managed in our emergency department, despite the limited safety profile available in the literature.

## 2 Case Report

### 2.1 Case History and Examination

**FIGURE 1:**
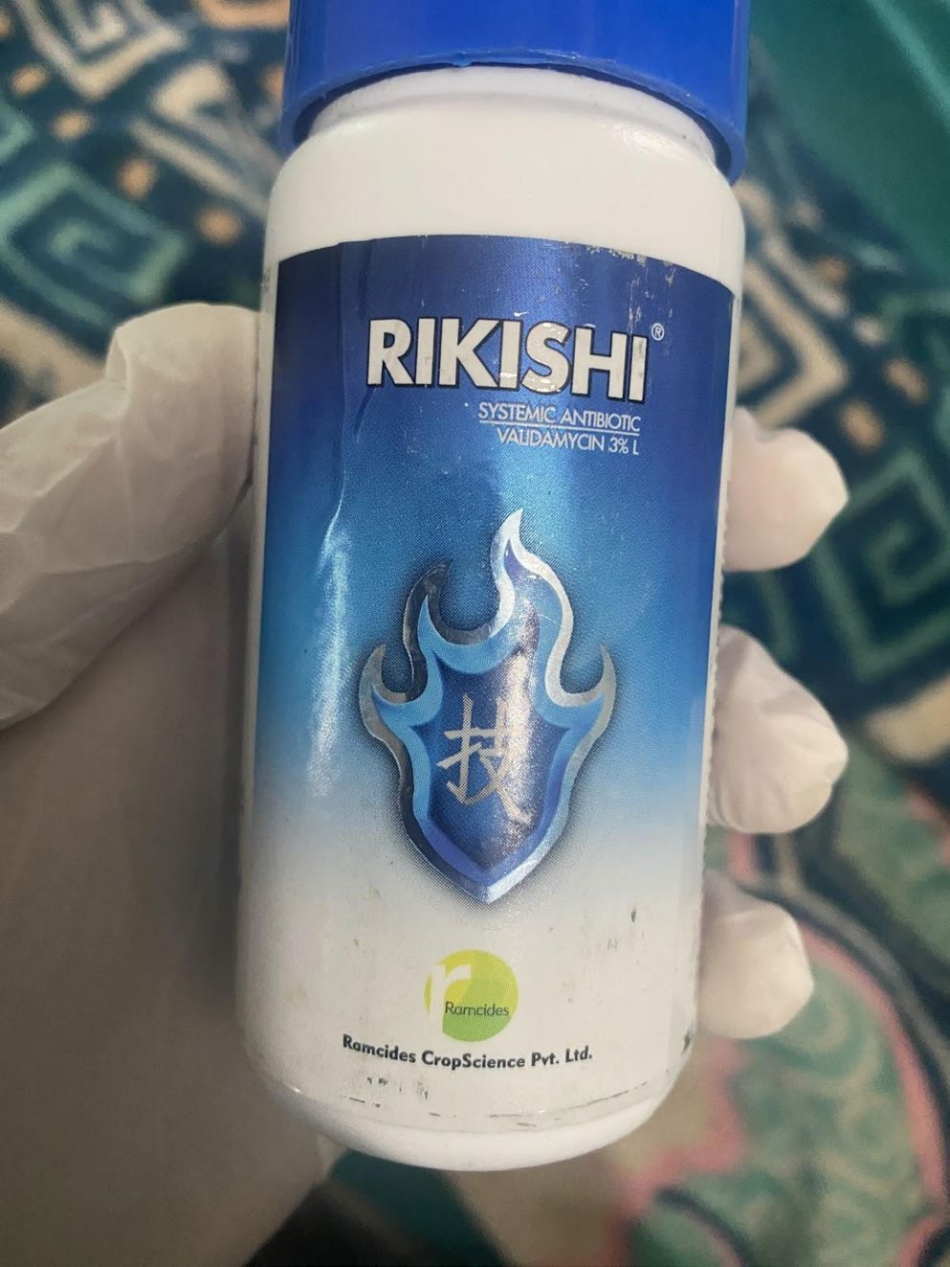
Bottle of Validamycin ingested by the patient.

A 32‐year‐old married female was brought to our hospital's emergency department within an hour of ingestion of 100 mL of 3% Validamycin, a fungicide used on agricultural lands (Figure 1). She presented with chief complaints of nausea, vomiting, and a burning sensation in the abdomen. On examination, she was afebrile and hemodynamically stable with a pulse of 126 beats per minute and blood pressure of 100/70 mmHg. Oxygen saturation on room air was 88%, for which she was given supplemental oxygen with nasal prongs at 2 L/min and maintained a saturation of > 94%. After an hour, she did not require any supplemental oxygen, and she maintained saturation on room air. She was agitated but responded relevantly to our questions. Bilateral pupils were 3 mm, round, regular, and reactive. Chest was clear on auscultation, and her abdominal palpation revealed a soft and nontender abdomen. No signs of excess salivation, dry mouth, hot flushed skin, diarrhea, or abnormal body movements were noted.

### 2.2 Investigations and Treatment

Validamycin poisoning was unfamiliar to the emergency physicians. So, a quick literature search for any reported cases was conducted. However, no relevant references regarding its ingestion and management were found. Consequently, we approached the case by following the general principles of management of poisoning. The patient's clothing and jewelry were removed, and her skin was wiped with normal saline‐soaked gauze to avoid possible absorption from the skin. Then, gastric lavage was performed using 2 L of normal saline, which yielded clear gastric contents. Two wide‐bore peripheral IV cannulas were inserted.

Samples were drawn for baseline investigations. She was administered intravenous antiemetics, a proton pump inhibitor, and fluid replacement was initiated. The patient was kept on nil per os.

Baseline investigations showed the following results:

- Complete blood count: WBCs: 9600/cu.mm, Hemoglobin: 12.7 g/dL, Platelets: 113,000/cu.mm.
- Random blood sugar: 89 mg/dL.
- Liver function test: Serum albumin: 3.9 g/dL, total serum bilirubin: 1.4 mg/dL.
- Serum AST/ALT: 13/45 IU/L, serum ALP: 42 U/L.
- Renal function test: Serum creatinine: 0.7 mg/dL, serum urea: 17 mg/dL, serum sodium: 136 mmol/L, serum potassium: 4.3 mmol/L.
- Serum calcium: 8.8 mg/dL.
- INR: 1.1.
- ABG: pH: 7.39, pCO_2_: 39 mm of Hg, pO_2_: 81 mm of Hg, Bicarbonate: 22 mEq/L.
- Chest X‐ray: Normal.

After initial management, we suggested referral to a higher center, as our hospital is not equipped with an intensive care setup that may be required at any point. Despite counseling about her current status and possible complications, the patient's family insisted on continuing treatment in our center due to financial constraints.

Laboratory tests were repeated every 12 h for a period of 48 h, with no significant alterations from baseline. Continuous vital monitoring and input/output charting were done. Similarly, there were no signs of CNS depression, respiratory, and hemodynamic compromise.

After stabilization of the patient, psychiatry consultation was conducted. At the time of examination, she was calm, conscious, cooperative, and well‐oriented to time, place, and person. She was lying comfortably in bed in a decubitus of her choice. She was appropriately dressed, with well‐maintained grooming and self‐care. Her motor activity was normal, with no signs of catatonia, conversion disorder, dissociation, social withdrawal, or autistic features. Her speech was assessed and found to be normal in rate, quantity, volume, tone, rhythm, and flow.

She stated that she felt angry at the time of poison ingestion. She described being blinded by anger and unable to control her actions. The anger was triggered by a quarrel with her sister. However, at the time of the mental status examination, she reported her mood as fearful. Her fears mainly centered around the consequences of consuming the poison.

The stream, form, and content of her thoughts were normal, with no evidence of obsessions, phobias, or delusions. There was also no evidence of hallucinations or illusions. Her attention, concentration, judgment, intelligence, and abstract thinking were intact. Insight was present. This examination revealed that consumption of poison was due to acute stress and high suicidal intent. There was no psychiatric comorbidity.

## 3 Conclusion and Results (Outcome and Follow‐Up)

She was discharged after 48 h of continuous monitoring, stable vitals, normal systemic examination, and laboratory findings. She was advised on psychiatric follow‐up. She was compliant with her follow‐up schedule and was doing well in her daily activities. She showed improvement in her relationships with her family members and social interactions.

## 4 Discussion

Our hospital is located in the Terai region of Nepal, where people rely on agriculture for their livelihood. Farmers use pesticides for pest control, making them easily accessible. As a result, we frequently encounter patients with poisoning in our emergency department, with organophosphorus compounds being the most common culprit [3, 4].

Sometimes, we also deal with uncommon compounds like Validamycin. It is an aminoglycoside antibiotic with antifungal properties, obtained from *Streptomyces hygroscopicus* var. limoneus. It is used in fields to treat sheath blight disease caused by Rhizopus [5, 6]. Trehalose is a non‐reducing disaccharide composed of two glucose molecules, which is present in high concentration in hemolymph (blood) sugar in insects [7]. Validamycin is a competitive inhibitor of trehalase that inhibits the conversion of trehalose to glucose, a property that has been used against a variety of organisms such as insects, nematodes, and fungi [8]. According to the World Health Organization's Recommended Classification of Pesticides by Hazard (2019), Validamycin belongs to category U, meaning “Unlikely to present acute hazard in normal use”. Its LD50 is 10,000 mg/kg body weight [9]. This means that large doses of Validamycin must be ingested for toxic manifestations to appear in humans, labeling it as practically non‐toxic. Clinical manifestations are not well established in humans. Diagnosis is solely based on the history of ingestion.

Enzymatic activity of trehalase has also been reported in the intestinal villi of humans, where it cleaves trehalose. Trehalase deficiency is a known disorder of carbohydrate absorption and transport, but its clinical domain is yet to be explored [10]. Thus, the effects of Validamycin on reducing trehalase activity or mimicking trehalase deficiency in humans could be a potential subject of study. To the best of our knowledge, there have been no reported cases of Validamycin toxicity in humans.

However, few cases have been reported in animal models. In a study by Fatma et al. on *Drosophila melanogaster* , it was found that at low doses (1– < 2.5 mM), Validamycin did not produce any significant toxicity, while at higher doses (5–10 mM), it had lethal effects. The study showed that Validamycin, in a dose‐dependent manner, causes Reactive Oxygen Species (ROS) buildup and DNA breaks in neuroblasts, a genotoxic effect that could potentially pose a risk in humans. However, the challenge lies in the fact that even acute high doses of Validamycin may not cause immediate toxic effects on humans. Yet, due to its widespread use, the potential neurotoxic effects from chronic exposure over time remain unknown, necessitating further research in the future [11].

According to a study published by Basnet A, Shrestha D, Chaulagain S et al., the mean age of poisoning from non‐organophosphates was 27.4 years, 69.8% were females, and 47.6% were married [12]. In our hospital, among all poisoning cases, the majority are young female adults. The intent of consumption is usually suicidal, even though most of them do not have a previous history of suicide attempt [12].

While the case aligns with the demographics of poisoning cases at our hospital, it also presents with a few peculiarities. The compound consumed is entirely new. The common triggers of suicidal poisoning in the community are interpersonal conflict, domestic violence, broken marriages, financial stress, and poor academic performance [13, 14, 15]. In this case, the trigger was an argument with her sister, which is a usual trigger in our setting. After a thorough psychiatric evaluation, no background of psychiatric illness was found. Thus, it raises a question of whether, in young individuals, an argument or a fit of rage can produce a high motive for suicide even in the absence of psychiatric illness.

A scientific approach to managing poisoning patients includes decontamination, administration of specific antidotes, and symptomatic management with continuous monitoring. In our case, symptomatic management was done with intravenous fluids, proton pump inhibitors, and antiemetics. In poisoning, it is always advisable to admit the patient and ensure continuous monitoring. The advantages of continuous monitoring are recognition and prompt management of delayed manifestations, monitoring of patient status, and detailed physical and psychiatric evaluations. Additionally, co‐ingestion of other substances should always be considered, as one poison may mask the symptoms of another. Repeated patient interviews and psychiatric assessments are recommended to obtain a thorough history.

From our experience managing this case of Validamycin ingestion, along with the review of available literature, we conclude that Validamycin is practically non‐toxic to humans. However, close monitoring is still required for the detection of co‐ingestions. Existing literature on poisoning in Nepal focuses primarily on medical aspects, often neglecting psychiatric concerns [12]. Our purpose in writing this article is to enhance awareness among healthcare professionals who work in resource‐limited settings.

Similarly, for the policymakers, this case serves as an important example of the need to maintain poison registries to document cases involving new compounds. We also recommend that emergency departments maintain a list of low‐toxicity substances to aid in prompt decision‐making and management.

It is beneficial to closely monitor the patient after discharge by healthcare providers and caregivers to avoid potential future suicidal attempts.

For emergency physicians working in rural, resource‐limited settings, this case is a good example to demonstrate that close observation and strict adherence to principles of management of poisoning can lead to complete recovery in cases involving less toxic compounds like Validamycin. In Low Middle Income Countries, avoiding unnecessary referrals helps conserve resources in tertiary care centers, ensuring their availability for other critically ill patients. Furthermore, it spares patients from financial burdens, which can be a significant stressor in low‐ and middle‐income countries.

Our study has few limitations; we could not confirm the diagnosis of validamycin ingestion through toxicological analysis. Also, it was not possible for us to measure the serum levels of Validamycin during the monitoring of patients, and the facility is not available in the country as well. Similarly, the lack of long‐term follow‐up of the patient and only one study limits the generalization of clinical findings. We believe future research and study will explore and give further clarifications.

## 5 Conclusion

Management of acute poisoning cases in hospital emergencies is a challenge in itself, but it becomes even more complicated when little is known about the ingested compound. In our case, the compound was Validamycin. The key to successful management was regular vitals and organ function monitoring, and symptomatic management. For comprehensive management of poisoning, one should go beyond the nature of ingestion of a compound, but rather focus on the intention behind the ingestion of the compound. Avoiding unnecessary referrals for less toxic compounds like Validamycin conserves human and financial resources, which is vital in low‐ and middle‐income countries like Nepal. Also, maintaining a poison registry can improve understanding and documentation of rarely encountered substances.

## Author Contributions

**Anish Luitel:** conceptualization, writing – original draft, writing – review and editing. **Sweekar Dahal:** conceptualization, writing – original draft, writing – review and editing. **Kabita Yadav:** writing – review and editing. **Bodhraj Budhathoki:** writing – review and editing. **Chandan Chaudary:** writing – review and editing. **Rasmita Poudel:** writing – original draft, writing – review and editing.

## Ethics Statement

Ethical approval is not required for publishing the case report at our hospital.

## Consent

A well written informed consent was obtained from the patient and her husband for publication.

## Conflicts of Interest

The authors declare no conflicts of interest.

## Data Availability

The data supporting the content of this article will be made available by the authors on request.
